# Aptamers in Bordeaux, 24–25 June 2016

**DOI:** 10.3390/ph10010014

**Published:** 2017-01-20

**Authors:** Jean-Jacques Toulmé, Paloma H. Giangrande, Günter Mayer, Beatrix Suess, Frédéric Ducongé, Bruce Sullenger, Vittorio de Franciscis, Fabien Darfeuille, Eric Peyrin

**Affiliations:** 1 ARNA Laboratory, Inserm U1212, CNRS UMR5320, University of Bordeaux, Bordeaux 33076, France; fabien.darfeuille@inserm.fr; 2 Department of Internal Medicine, University of Iowa, Iowa City, IA 52242, USA; paloma-giangrande@uiowa.edu; 3 Unit Chemical Biology and Medicinal Chemistry, University of Bonn, Bonn 53121, Germany; gmayer@uni-bonn.de; 4 Department of Biology, Technical University Darmstadt, Darmstadt 64287, Germany; bsuess@bio.tu-darmstadt.de; 5 CEA, DRF, I2BM, MIRCen, CNRS UMR 9199, University Paris Saclay, Fontenay aux Roses 92265, France; frederic.duconge@cea.fr; 6 Department of Surgery, Duke University, Durham, NC 27710, USA; bruce.sullenger@duke.edu; 7 Institute of Experimental Endocrinology and Oncology, CNR, Naples 80145, Italy; defranci@unina.it; 8 Department of Molecular Pharmacochemistry, CNRS UMR5063, University Grenoble Alpes, Saint Martin d’Hères 38400, France; eric.peyrin@univ-grenoble-alpes.fr

**Keywords:** aptamer, in vitro selection, SELEX, riboswitch, oligonucleotide, biosensor, cardiovascular, cancer, cell-targeted aptamers, imaging, synthetic biology

## Abstract

The symposium covered the many different aspects of the selection and the characterization of aptamers as well as their application in analytical, diagnostic and therapeutic areas. Natural and artificial riboswitches were discussed. Recent advances for the design of mutated polymerases and of chemically modified nucleic acid bases that provide aptamers with new properties were presented. The power of aptamer platforms for multiplex analysis of biomarkers of major human diseases was described. The potential of aptamers for the treatment of cancer or cardiovascular diseases was also presented. Brief summaries of the lectures presented during the symposium are given in this report. A second edition of “Aptamers in Bordeaux” will take place on September 2017 (http://www.aptamers-in-bordeaux.com/).

## 1. Aim and Scope of the Symposium

Twenty-six years after the publication of three seminal papers on in vitro selection of ligands and enzymes from randomly synthesized oligonucleotide libraries, aptamers are coming to fruition. From these early days, new methodologies have been described, beyond SELEX—the gold standard—for the identification and the optimization of aptamers that can be subsequently converted into biotechnological tools or integrated into various devices. Health and life sciences are still major areas of investigation; indeed, diagnostic and therapeutic applications of aptamers drive the activity of many laboratories and companies. However, other areas (the agro-food industry, environment, etc.) take advantage of aptamer characteristics for analytical purposes. Most of these aspects were covered by about 100 communications presented during the eight sessions of the symposium: riboswitches, selection methodology, aptamer chemistry, biosensors, diagnostics, therapeutics and clinical trials. Over 160 participants—biologists, biophysicists, chemists, engineers, clinicians—either from academy or from industry, coming from 17 countries, contributed to this very stimulating interdisciplinary symposium. Furthermore, this meeting offered the opportunity for 44 PhD students to present their work. The program included not less than six keynote lectures and 26 invited oral presentations which are briefly summarized in this report.

## 2. Conferences

### 2.1. Riboswitches

The first session of the meeting chaired by Dr. Fabien Darfeuille (University of Bordeaux, Bordeaux, France) was a joint session co-organized with the Bordeaux RNA club symposium and focused on two exciting research domains linking the aptamer field with RNA biology, namely riboswitches and ribozymes. Dr. Dominique Burnouf (Institut de Biologie Moléculaire et Cellulaire, Strasbourg, France) introduced the folding dynamics of RNA regulation mediated by two bacterial TPP riboswitches (one involved in the transcriptional control of ThiC and one involved in the translational control of ThiM in *Escherichia coli*) and one eukaryotic TPP riboswitch regulating alternative splicing in plants (*Arabidopsis thaliana*). By using a new technique named kinITC, developed in his laboratory, he showed that in contrast to the TPP riboswitch found in plants, the two bacterial TPP riboswitches are kinetically controlled. This regulation requires that the RNA polymerase pauses after synthesis of each riboswitch aptamer to leave time for TPP binding, but only when its concentration is sufficient for binding. Dr. David Horning (Scripps Research Institute, La Jolla, CA, USA) described the use of directed evolution to improve the activity of an RNA polymerase ribozyme by selecting variants that can synthesize functional RNA aptamers from an RNA template. Beyond the interest of this work with respect to the RNA-based origin of life, it also shows the potential use of this ribozyme to replicate and amplify short RNA molecules in a way similar to the polymerase chain reaction (riboPCR).

The keynote speaker of the first session was Dr. Pascale Cossart (Institut Pasteur, Paris, France). She first started her lecture by playing a movie describing at the molecular level the life cycle of the main bacterial pathogen responsible for foodborne infections, *Listeria monocytogenes*. She briefly revisited all the work done on this model organism regarding RNA-based regulation and, in particular, the role played by a specific RNA stem-loop structure (RNA “thermometer”) involved in the activation of virulence genes in response to a temperature shift. She introduced several examples dealing with the regulation mediated by antisense RNA and/or overlapping operons and presented the excludon concept. In this context, she then showed that riboswitches are not only controlling the expression of mRNAs but are also controlling the transcription of noncoding RNAs. In particular, she showed the importance of such regulation in controlling ethanolamine utilization in response to B12, a vitamin required as a co-factor in this metabolic pathway. She finally presented her latest work done in collaboration with Dr. Rotem Sorek and the development of a new approach named Term-seq. Using this new technology, they discovered numerous antibiotic-responsive ribo-regulators that control antibiotic resistance genes in bacterial pathogens. As an example, she described the existence of an attenuation mechanism mediated by antibiotic-stalled ribosomes in *L. monocytogenes*.

### 2.2. Nucleic Acid Ligands and Switching Aptamers

The second morning session of 24 June, chaired by Prof. Eric Peyrin (University Grenoble-Alpes, Grenoble, France), was dedicated to small chemical ligands binding to RNA that could induce conformational changes or perturb the biological function of an RNA sequence. Dr. Jean-Jacques Toulmé (University of Bordeaux, Bordeaux, France) described biosensors based on the conditional formation of kissing complexes in response to a conformational change of aptamers induced by their cognate ligand. So-called aptaswitches were engineered for the specific recognition of purine analogues: adenosine, theophylline and GTP. For multiplex detection of these three molecules either by surface plasmon resonance in a grafted format by fluorescence, Dr. Toulmé took advantage of a large repertoire of RNA-RNA, RNA-DNA and DNA-DNA kissing motifs. He described the use of aptaswitches for driving the assembly of oligonucleotide scaffolds through kissing interactions. In the second lecture of this session, Dr. Maria Duca (Institute of Chemistry, Nice, France) reported on a series of synthetic RNA ligands aimed at preventing the maturation of oncogenic pre-microRNA by DICER. A library of 640 compounds was screened in vitro, allowing the identification of molecules of interest. The best compound inhibited the proliferation of cells over-expressing targeted miRNAs in a specific manner. This effect was correlated to the decrease in the production of oncogenic miRNAs as shown by qRT-PCR and luciferase reporter experiments and to the de-repression of miRNA target proteins. The lecture by Prof. Kazuhiko Nakatani (Institute of Scientific and Industrial Research, Osaka, Japan) was dedicated to the regulation of gene expression by ligands inducing a −1 ribosomal frameshifting. Small molecules were designed to bind to mismatched regions in double-stranded RNA (or DNA). Naphthyridine carbamate tetramer (NCTn), one of these molecules, strongly stabilizes mismatched duplexes containing a guanine-rich sequence. Prof. Nakatani described that the NCTn-inducible formation of an RNA pseudoknot can regulate the translation of a target reporter gene by −1 ribosomal frameshifting both in vitro and in cultured cells. Dr. Heiko Sievers (Dynamic Biosensors GmbH, Martinsried, Germany) presented the switchSENSE biochip technology that employs DNA nanolevers for real-time measurement of thermodynamic and kinetic parameters of interactions. He demonstrated the potential of this technology for protein-DNA complexes.

### 2.3. Synthetic Genetics and XNA Aptamers

The Saturday afternoon session, chaired by Dr. Vittorio de Franciscis (CNR, Naples, Italy), started with the presentation of Dr. Thomas Shubert (2bind GmbH, Regensburg, Germany) about an innovative method to analyze biomolecular interactions in solution at the microliter scale. MicroScale thermophoresis (MST) is a technique based on the analysis of the movement of molecules through temperature gradients and is easily applicable to measuring interactions between aptamers and their targets. MST assays are provided by 2bind GmbH. The following three scientific lectures were focused on the possibility of expanding the functional potential of nucleic acids by increasing the chemical diversity of functional groups or increasing their stability in serum. Increasing the bio-stability and target interactions of aptamers are topics of particular interest for in vivo applications. In the past decade, many groups have suggested adding functionality to the four standard DNA and RNA building blocks to obtain more efficient aptamers. The first lecture given by Dr. Elisa Biondi (FfAME, Alachua, FL, USA) reported research focused on the possibility of adding replicable nucleotides to the DNA alphabet, creating an artificially expanded genetic information system (AEGIS) by developing a new molecular biology to support this expanded laboratory in vitro evolution (LIVE) process and the analytical chemistry needed to sequence AEGIS DNA survivors that might emerge under selective pressure. The speaker presented data from examples of this process in three successful AEGIS-LIVE experiments, two of which were targeted towards whole-living cancer cells, and one towards a specific protein target, the protective antigen from *Bacillus anthracis*. The following talk by Dr. Rihe Liu (University of North Carolina, NC, USA) reported the development of a robust system that allows the direct selection, with high efficiency, of aptamers from a 2′ fully modified fGmH RNA (2′-F-dG, 2′-OMe-dA/dC/dU) library transcribed by a variant of the T7 RNA polymerase. Results revealed the higher stability of fGmH RNA aptamers against alkaline hydrolysis, as well as resistance to many of the nucleases present in serum, as compared to 2′ partially modified RNA variants. Further, Dr. Liu reported that the resulting aptamers exhibit high target binding affinity and specificity, thus potentially improving their in vivo applicability as therapeutics and/or as diagnostic tools. The keynote lecture was given by Prof. Philipp Holliger (MRC Laboratory of Molecular Biology, Cambridge, UK) who closed the session by providing a framework of the extraordinary recent progresses achieved in the last few years exploring the chemical etiology of nucleic acid evolution. Being conceivable that the variety of nucleobases was larger than RNA and DNA at the initial steps of evolution, he presented data on the novel synthetic nucleic acid polymers (XNAs) with fully altered backbone chemistries in which the canonical five-membered ribofuranose ring of DNA and RNA has been replaced by non-natural congeners. Prof. Holliger showed that eight different synthetic polymers, based on nucleic acid architectures, can also mediate genetic information storage and propagation. He demonstrated a capacity for Darwinian evolution by the de novo selection of specific ligands (XNA aptamers) and catalysts (XNAzymes) based on entirely synthetic backbones, and by the in vitro evolution of RNA polymerase ribozymes able to catalyze the synthesis of RNA polymers exceeding their own size.

The last session on Friday, chaired by Dr. Günter Mayer (University of Bonn, Germany), included four talks in different aptamer fields ranging from improved selection methodologies to nanostructure and biosensor applications. The first speaker of the session, Dr. Ichiro Hirao (Institute of Bioengineering and Nanotechnology, Singapore), focused his talk on the genetic alphabet expansion technology that uses artificial base pairs with different chemical and physical properties to improve the affinity of DNA aptamers. Through the use of the unnatural base pair between 7-(2-thienyl)imidazo[4,5-b]pyridine and 2-nitro-4-propynylpyrrole, DNA aptamers with enhanced affinity were described for various targets including VEGF165, interferon-γ and vWF. Furthermore, Dr. Hirao presented an aptamer stabilization strategy relying on the introduction of a nuclease-resistant mini-hairpin structure into the interferon-γ DNA aptamer, protecting about 70% of this aptamer in human serum (at 37 °C after three days). Next, Dr. Curtis H. Lam (AM Biotechnologies, Huston, TX, USA), described the non-SELEX bead-based selection of chemically modified aptamers directed against the snake venom myotoxin. The dissociation constant determined by biolayer interferometry was found to be about 40 nM for the highest-affinity sequence. Dr. Lam subsequently reported the results of in vivo anti-hind limb paralysis assays in mice. The addition of 16 equivalents of aptamer resulted in a complete neutralization of the symptoms of the myotoxin, establishing the proof of principle for the use of aptamers as a possible treatment of snakebite. Dr. Chenze Lu (University Grenoble-Alpes, France) gave a presentation on the use of the split-aptamer strategy to construct DNA assemblies mediated by the presence of a small ligand, i.e., adenosine. Surface plasmon resonance was used to evaluate the ability of the adenosine molecule to trigger the formation of a multiple chain structure. The experimental conditions such as the strand concentration, flow rate and grafting density of probes were optimized to provide a significant adenosine-based SPR signal. The last speaker of this day, Prof. Eric Peyrin (University Grenoble-Alpes, France), described the recent developments in the fluorescence anisotropy–based aptamer sensing area. The main design strategies and transduction principles were detailed and discussed in relation to the kinds of targets and fluorophores. Some detection examples in real samples were also reported in order to demonstrate the potential applicability of the reported methodologies.

### 2.4. Biological Applications of Aptamers and Aptamer Platforms

Saturday morning’s session, chaired by Dr. Paloma H Giangrande (Iowa University, Iowa, USA), included four lectures covering various topics ranging from novel approaches to SELEX (systematic evolution of ligands by exponential enrichment) to new aptamers and applications of aptamers for diagnostics. The first speaker of the session, Dr. Günter Mayer (University of Bonn, Germany), reported on adaptive dynamic artificial poly-ligand targeting (ADAPT) as an ssDNA aptamer-based highly multiplexed biomarker discovery platform. The ADAPT platform identifies and measures thousands of ODNs that form signatures by their association with differentially expressed biomolecules in their native states. Dr. Mayer presented data in breast cancer suggesting that ADAPT may have a promising utility as a breast cancer diagnostic tool that can be easily extended to developing diagnostics for other malignancies. The potential of this ADAPT platform was further described for exosome profiling by Dr. D.B. Spetzler (Caris Life Sciences, Phoenix, AZ, USA). Dr. Henning Ulrich (University São Paolo, Brazil) reported on DNA aptamer ligands, which bind to proteins secreted into the erythrocyte plasma membrane by the malaria-causing parasite *Plasmodium falciparum*. This parasite causes hundreds of millions of cases of malaria and is responsible for more than a million deaths of children per year alone in Africa. Dr. Ulrich’s research group is currently using these high-affinity aptamers for isolation and identification of their parasite target proteins. Future applications of these aptamers will include novel probes for malaria imaging and diagnostics and new drugs for potential therapeutic applications. Dr. O’Sullivan (Universitat Rovira I Virgili, Tarragona, Spain) reported on two ultrasensitive aptamer-based assays for the detection of β-conglutin. The two approaches were compared in terms of the detection limits and assay time required, and can be adapted (by modifying the aptamer) for the detection of other clinically relevant targets. Dr. Larry Gold (SomaLogic, Inc., Boulder, CO, USA) gave a keynote presentation on a novel class of aptamers called SOMAmers (which are slow off-rate modified aptamers), developed at SomaLogic, Inc. Using their SOMAmer-based technology (SOMAScan), Dr. Gold and his team were able to simultaneously quantify over 4000 human proteins in various sample matrices including blood, urine, tissues, and more. To date, they have analyzed over 100,000 human blood samples from a variety of physiological and/or medical conditions—heart disease, Alzheimer’s disease, Duchenne muscular dystrophy, several cancers and diet studies. Dr. Gold concluded his presentation by presenting to the audience what he referred to as the ‘Big Dream at SomaLogic The Wellness Chip’—an ambitious undertaking at SomaLogic to develop a chip capable of monitoring the health profile of every individual over time: the future of personalized medicine.

### 2.5. Aptamer-Based Probes for Imaging

The second session on Saturday, chaired by Dr. B. Sullenger, started with the TRAIL lecture given by Dr. Samie R. Jaffrey (Cornell University, New York, NY, USA). He reported about the selection and utilization of RNA aptamers that bind fluorophores and image RNA in living cells. The aptamers bind small-molecule dyes that become fluorescent only upon specific binding to the aptamer. These fluorescent switches are excellent tags for imaging RNA molecules inside cells. Dr. Jaffrey introduced several aptamer variants termed Spinach, Corn and Red Broccoli which offer a whole spectrum of fluorescent colors analogous to green and red fluorescent proteins. He demonstrated the use of these aptamers as genetically encoded real-time biosensors for cellular metabolites and proteins. For this, a metabolite-sensing RNA (e.g., the TPP-binding riboswitch) was fused to the Spinach aptamer, allowing fluorescence-based quantification of the metabolite level in the cell. In the second talk of the session, Dr. Beatrix Suess (TU Darmstadt, Darmstadt, Germany) discussed several strategies for the use of small-molecule binding aptamers for the conditional control of gene expression such as translation initiation, mRNA splicing or mRNA stability and they were shown to efficiently function in all three domains of life. Dr. Frédéric Ducongé (CEA MIRCen, Fontenay aux Roses, France) presented PATTERNITY-Seq, a method to improve the identification of aptamers using next-generation sequencing. The method builds dendrograms (named FREDrograms) to visualize the evolution of sequences and their family relationship at each round of SELEX. Re-analyzing data from a cell-SELEX using this approach, improved aptamers were identified against targets such as Annexin-2 compared to those previously published. Finally, Dr. Anna Zamay (Krasnoyarsk State Medical University, Krasnoyark, Russia) presented the ex vivo selection of aptamers against human glioblastoma. These aptamers were conjugated through a biotin/streptavidin linkage to a Brilliant Violet 650 dye and evaluated on freshly resected tumors using a surgical fluorescence microscope. The goal of this approach, named AptaMargin, is to improve the ability of neurosurgeons to specifically and completely resect human brain tumors using in situ fluorescent visualization of tumor margins.

### 2.6. Pre-Clinical and Clinical Applications of Aptamers

In the session chaired by Dr. F. Ducongé (CEA MIRCen, Fontenay aux Roses, France), Dr. Maxim Berezovski (University of Ottawa, Canada) described an original application of aptamers targeting the VSV (vesicular stomatitis virus) oncolytic virus in order to hide them from neutralizing antibodies. The goal is to protect them from being cleared in the bloodstream before they reach tumor cells and exert their effect. Alternatively, aptamers were raised against the Fab fragment of anti-VSV polyclonal antibodies. These aptamers were then engineered as multivalent binders. The most effective constructs increased the viral infectivity by 70% in vitro. Another application in the field of immunology was described by Dr. Jean Gariépy (University of Toronto, Canada): he designed a pegylated aptamer raised against a CD200R1 target that blocks inflammatory responses in mouse models. Importantly, this aptamer does not suppress cytotoxic T-lymphocyte induction in lymphocyte culture. Dr. H. Tom Soh (Stanford University, Stanford, CA, USA) described real-time biosensor-integrating aptamers in a micro-fluidic device capable of tracking circulating molecules in living mice. He presented the results obtained for monitoring a cancer drug (doxorubicin) and an antibiotic (kanamycin). This device can also be used for feedback control of drug concentration.

Saturday afternoon’s session chaired by Dr. Beatrix Suess (TU Darmstadt, Darmstadt, Germany) included five lectures on novel therapeutic applications of aptamers. The first speaker of the session, Dr. Paloma Giangrande (University of Iowa, Iowa City, IA, USA), reported on cell-targeted RNA aptamers directed against vascular smooth muscle cells. Dr. Giangrande demonstrated that these aptamers have superior therapeutic effects when compared to the gold-standard drug-eluting stents (stents coated with general growth inhibitors); the aptamers inhibit the pathologic proliferation of vascular smooth muscle cells without affecting the healing of the endothelial cell layer (re-endothelialization). Dr. Giangrande discussed how cell-targeting aptamers can be developed as therapeutics for many different diseases including cancer and diabetes, where it is beneficial to limit undesired toxic effects of the drug to normal cells. Dr. Ulrich Hahn (University of Hamburg, Germany) gave a nice overview of his aptamer program over the years and discussed his recent focus on the development of aptamers that inhibit cell adhesion. He provided examples of high-affinity DNA aptamers that target E- and P-selectin and demonstrated the inhibitory effects of these aptamers in cancer cells in culture. Dr. Hahn also touched on ongoing new projects aimed at the selection of aptamers specific for further components playing a central role in the promotion of tumor cell growth, invasion, and organotropic metastasis, such as integrins. Dr. Sullenger (Duke University, Durham, NC, USA) reported on the therapeutic and diagnostic applications of rapidly reversible aptamers. He discussed the development and clinical evaluation of the factor IXa aptamer. This aptamer was recently evaluated in 2000 patients undergoing angioplasty. Although the aptamer-antidote pair failed due to safety issues with the formulation, it demonstrated that aptamers can rapidly and potently inhibit their target proteins in patients and that antidote molecules can rapidly reverse such activity in the minute time frame. Based on these initial promising results with the aptamer-antidote technology, Dr. Sullenger’s group is exploring the potential of using antidote-mediated control of aptamers for a variety of other therapeutic and diagnostic applications. One application includes the use of a rapidly controllable factor Xa anticoagulant aptamer to effectively control (reverse) blood coagulation during cardiopulmonary bypass surgery. A second application involves a rapidly reversible thrombin aptamer for imaging blood clots in real time. Finally, the group is also developing a rapidly reversible tumor-targeting aptamer to limit the side effects of chemotherapeutic agents. Dr. Brett Schrand (University of Miami, USA) reported on the use of aptamers as an adjuvant to radiation therapy. He discussed the application of VEGF-targeted 4-1BB co-stimulation of T-cells to enhance radiation-induced immune control of tumor growth using a multimeric aptamer approach linking two aptamers (one to VEGF and the other to 4-1BB). Dr. Schrand reported efficacy in several animal models of cancer. For example, he showed that treatment of tumor-bearing mice with VEGF-4-1BB aptamer conjugates was effective against melanoma, breast cancer, fibrosarcoma and glioma. He went on to show that this effect was greatly enhanced with radiation, through the activation of VEGF following radiation treatment. Finally, he presented key findings that validated this approach in a model for abscopal responses and showed that VEGF-targeted 4-1BB costimulation was effective at inducing an immune response against a distant, non-irradiated tumor, comparable to that of CTLA-4 and/or PD-1, the gold standard in tumor immunotherapy, without the off-target effects associated with these therapies. Dr. Vittorio de Franciscis (Institute of Experimental Endocrinology and Oncology, CNR, Naples, Italy) reported on aptamer carriers for cancer stem cell–targeted delivery of therapeutic miRNAs for the treatment of glioblastiomas (GBM). GBM is the most common primary brain tumor and one of the most lethal kinds of cancer sustained by a small population of cancer stem cells (glioblastoma stem cells, GSCs). Recent interest has focused on the use of miRNAs and miRNA inhibitors (antimiRs) for the treatment of this disease. However, a major obstacle to the clinical translation of this new class of oligonucleotide drugs is the lack of a reliable targeted delivery method. Dr. de Franciscis reported on exciting work from his lab demonstrating the use of aptamers targeted to glioblastoma cells as potential delivery agents for therapeutic miRNAs and miR inhibitors. In addition, he presented evidence that when used in combination, these aptamer-miR conjugates result in enhanced therapeutic effects, leading to the suppression of tumor sphere formation. Results provide the rationale for the design of combined aptamer-miR–based gene therapies selectively targeting cancer stem cells.

## 3. Conclusions—Poster Awards

To highlight the remarkable work carried out by young investigators, two out of 69 posters were selected for awards. The first award was given to Nam Nguyen Quang, a PhD student supervised by Dr. Frédéric Ducongé (CEA MIRCen, Fontenay aux Roses, France), who presented PATTERNITY-seq, a new bioinformatics pipeline to analyze every SELEX round using next-generation sequencing. This method monitors the paternity relationship and the evolution of several patterns from primary sequences to predicted secondary structures. It can help to identify aptamer families and the best aptamers of a family. The second award was given to Marc Vogel, a PhD student in the laboratory of Dr. Beatrix Suess (TU Darmstadt, Darmstadt, Germany), who introduced a recently developed synthetic riboswitch which allows the control of alternative splicing in mammalian cells. Using the tetracycline-binding aptamer, a control device was developed which regulates the accessibility of the 3′ splice site, leading to exon skipping or the lack thereof. The functionality of this device was demonstrated in different host genes and cell lines including a suicide system that allows efficient, tetracycline-dependent induction of cell death via the controlled expression of a CD20 surface receptor.

Following the success encountered by this symposium, a new edition will be organized in Bordeaux on 22–23 September 2017 (http://www.aptamers-in-bordeaux.com/) as a satellite of the annual meeting of the “Oligonucleotide Therapeutic Society” (24–27 September 2017).

